# Adverse childhood experiences and non-suicidal self-injury in adolescents: the roles of depressive symptoms and teacher care

**DOI:** 10.3389/fpsyt.2026.1790188

**Published:** 2026-05-04

**Authors:** Juan Guo, Chunhui Qi, Xiangyan Li, Xiaoyu Huang, Zhen Zhang

**Affiliations:** Faculty of Education, Henan Normal University, Xinxiang, China

**Keywords:** adolescents, adverse childhood experiences, depressive symptoms, non-suicidal self-injury, teacher care

## Abstract

**Background:**

Non-suicidal self-injury (NSSI) has become an increasingly prominent mental health problem during adolescence and often co-occurs with depressive symptoms, anxiety, personality-related difficulties, and experiences of childhood trauma, forming a complex psychosocial risk structure. From a risk and protective factor perspective, the present study examined the associations among adverse childhood experiences (ACEs), depressive symptoms, perceived teacher care, and NSSI among Chinese adolescents.

**Methods:**

The participants were 2,221 junior high school students from three schools in China. Data were collected using the Childhood Trauma Questionnaire, the Adolescent Non-Suicidal Self-Injury Questionnaire, a depression scale, and a teacher care scale.

**Results:**

Adverse childhood experiences and depressive symptoms were significantly positively associated with NSSI, whereas perceived teacher care was significantly negatively associated with NSSI. Higher levels of ACE exposure and more severe depressive symptoms were associated with higher levels of NSSI, while higher levels of teacher care were associated with lower levels of NSSI. Moreover, among adolescents reporting higher levels of perceived teacher care, the positive associations between ACEs and NSSI and between depressive symptoms and NSSI were attenuated, suggesting that teacher care, as an external support resource in the school context, may play an important protective role.

**Conclusions:**

Adverse childhood experiences and depressive symptoms are important risk factors for adolescent NSSI, whereas teacher care plays a significant protective role in the school context. School-based prevention and intervention efforts should prioritize adolescents with high levels of ACE exposure and pronounced depressive symptoms, while strengthening teacher care to enhance protective resources. Future research should further explore how specific dimensions and timing of ACEs, trajectories of depressive symptoms, and multiple sources of social support jointly influence the development and maintenance of NSSI in adolescents.

## 1 Introduction

Non-suicidal self-injury (NSSI) refers to the deliberate and repeated damage to one’s own body tissue in the absence of explicit suicidal intent, with common forms including cutting, hitting, and burning (1). Unlike suicidal behavior, the primary motivation for NSSI lies in alleviating intense negative emotions or achieving emotional release (2). NSSI is widespread among adolescents and tends to peak between the ages of 15 and 17 (3). Research indicates that the lifetime prevalence of NSSI among adolescents worldwide can reach 28.2% (4). In recent years, the incidence of NSSI has shown an upward trend globally, making it a serious public health concern (3). Studies conducted among Chinese adolescents have reported that 17.38%–29% have a history of NSSI (5–7). NSSI is closely associated with various psychological disorders, including depression, anxiety, and posttraumatic stress disorder, and is considered an important predictor of suicidal ideation and suicide attempts (8, 9). Therefore, systematically identifying risk and protective factors associated with adolescent NSSI is of substantial practical significance.

Adverse childhood experiences (ACEs) typically refer to negative life events occurring before the age of 18, such as abuse, neglect, exposure to violence, emotional deprivation, or family dysfunction (10), and have been identified as important distal risk factors for NSSI (5, 11, 12). The developmental psychopathology framework suggests that early traumatic experiences may disrupt the development of emotion regulation and interpersonal competence, thereby increasing the likelihood of adopting maladaptive coping strategies such as self-injury (13). The integrated model of NSSI further proposes that childhood maltreatment, as a distal vulnerability factor, may increase the tendency to engage in NSSI as a means of regulation when individuals encounter peer conflict or intense negative emotions (14). In the present study, ACEs were operationalized using the Childhood Trauma Questionnaire (CTQ), which primarily captures abuse- and neglect-related adversity. These dimensions represent core forms of early adversity that have been consistently linked to emotional maladjustment and maladaptive coping behaviors, including non-suicidal self-injury (NSSI). Empirical studies have consistently shown that adolescents who have experienced abuse or neglect are more likely to engage in NSSI (12, 15, 16).

Although the risk association between ACEs and NSSI has been well documented, the emotional processes underlying this relationship remain to be further clarified. Depressive symptoms are among the most central emotional risk indicators in the field of affective disorders; they are both common outcomes of ACEs and highly co-occurring with NSSI, and depression may play a key role in the onset and maintenance of NSSI (5, 17–19). The experiential avoidance model (EAM) proposes that NSSI functions as a means to escape or alleviate the distress associated with negative emotions and traumatic memories. When individuals experience negative affect, NSSI may provide temporary emotional relief, thereby forming a negative reinforcement cycle that promotes the repetition of self-injurious behavior; in essence, NSSI serves an emotion avoidance function (20). As a persistent negative emotional state, depression has been identified as an important psychological risk factor for NSSI (5, 21). Research has shown that using NSSI to alleviate psychological distress associated with depression becomes a coping strategy repeatedly relied upon by adolescents who self-injure when facing emotional difficulties (18, 22). Moreover, individuals with histories of ACEs tend to exhibit more severe depressive symptoms and a higher propensity for NSSI (23). These findings suggest that depressive symptoms are likely to constitute an important proximal emotional risk pathway in the association between ACEs and NSSI.

Although ACEs and depressive symptoms are associated with elevated NSSI risk, not all adolescents who experience childhood adversity engage in NSSI, indicating the presence of protective factors that may buffer this pathway. Teacher care, as an important source of social support in the school context, refers to students’ subjective perceptions of teachers’ understanding, responsiveness, fairness, and emotional support (24). According to the risk buffering hypothesis, when individuals are exposed to high-risk environments, the presence of positive external support can substantially mitigate negative outcomes (25). Empirical evidence suggests that teacher care, as a key source of social support within the school environment, can alleviate the negative emotional impact of adverse experiences by improving adolescents’ emotion regulation and coping strategies (26). Teacher caring behaviors also serve as important protective factors against academic stress among middle school students and have a direct influence on students’ emotional functioning (27, 28). According to the experiential avoidance model, NSSI may function as a maladaptive strategy for escaping or reducing intense negative emotional states (20). Adolescents with elevated depressive symptoms often experience persistent sadness, hopelessness, and self-critical cognition, all of which may increase the tendency to rely on avoidance-oriented coping strategies such as self-injury (22, 29). Teacher care may weaken this pathway by providing emotional validation, interpersonal safety, and supportive guidance, thereby reducing the need to use NSSI as a means of affect regulation. Supportive teacher–student relationships may enhance adolescents’ sense of school belonging and coping flexibility, helping them respond to depressive distress through more adaptive means (24, 26, 30, 31). Accordingly, teacher care may attenuate the association between depressive symptoms and NSSI.

Although the associations among ACEs, depressive symptoms, and NSSI have been widely examined, less is known about how school-based relational resources may operate within this framework. In particular, perceived teacher care has received relatively limited attention as a proximal protective factor in adolescents’ everyday school context. In addition, evidence from non-Western settings remains comparatively scarce. Therefore, the present study extends the literature by examining ACEs, depressive symptoms, teacher care, and NSSI simultaneously in a large sample of Chinese adolescents, with particular attention to the buffering role of teacher care in a culturally relevant school context.

In summary, based on a risk–protective factor framework and existing empirical evidence, the present study examined the relationships among adverse childhood experiences, depressive symptoms, teacher care, and non-suicidal self-injury in adolescents, and proposed the following hypotheses:

H1: Depressive symptoms play an indirect role in the association between adverse childhood experiences and NSSI.

H2: Teacher care moderates the association between adverse childhood experiences and NSSI, as well as the association between depressive symptoms and NSSI.

**Figure 1:**
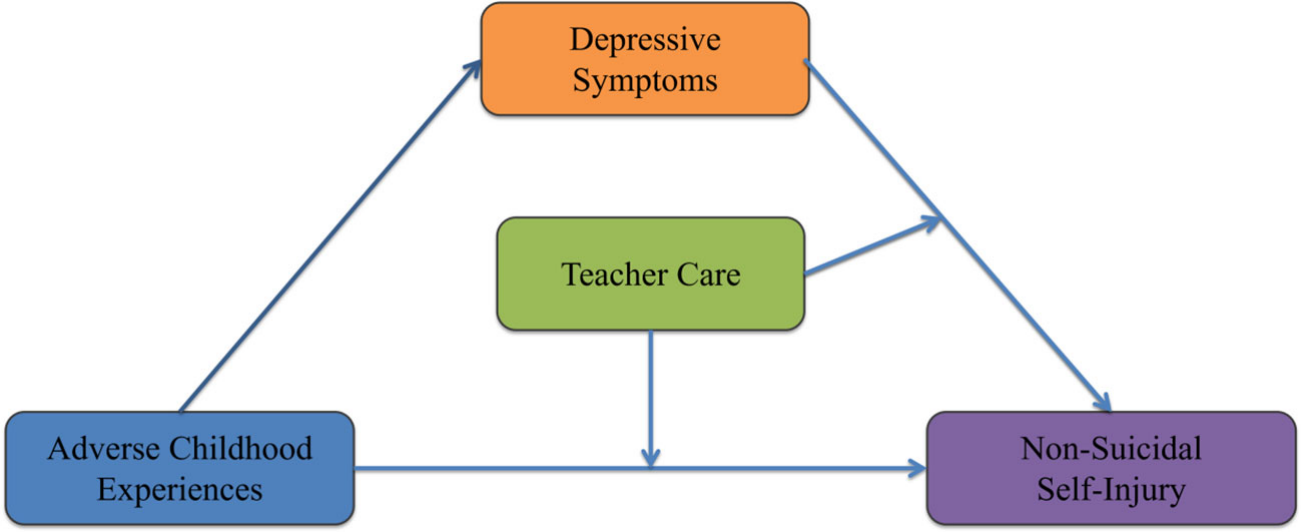
Hypothesized model.

The theoretical hypothesized model proposed in this study is presented in **Figure 1**.

## 2 Materials and Methods

### 2.1 Participants

This study employed a cross-sectional design and used stratified cluster sampling to recruit junior high school students from central China. Three schools were selected: an urban key middle school (approximately 1,000 students), a school primarily serving children of rural-to-urban migrant workers (approximately 1,100 students), and a rural middle school (approximately 300 students). Data were collected using a combination of online and paper-and-pencil surveys. In the urban and migrant schools, three trained psychology teachers administered the questionnaires in school computer rooms under standardized conditions; in the rural school, students completed traditional paper questionnaires in classroom settings. A total of 2,400 questionnaires were distributed. Based on predefined criteria including minimum completion time (≥ 300 seconds) and age range (12–16 years), data were screened for quality. After excluding invalid or ineligible responses, 2,221 valid questionnaires were retained, yielding an effective response rate of 92.54%.To further characterize the heterogeneity of the sample, one-way ANOVA was conducted to compare the three school types on the main study variables. Significant between-school differences were found for ACEs (*F* = 22.70, *p* <.001), NSSI (*F* = 5.46, *p* <.01), depressive symptoms (*F* = 4.16, *p* <.05), and teacher care (*F* = 26.61, *p* <.001).

### 2.2 Measurement tools

Childhood Trauma Questionnaire (CTQ): Adverse childhood experiences were assessed using the CTQ. In the present study, the CTQ total score was used to operationalize ACEs, primarily reflecting abuse- and neglect-related childhood adversity. The CTQ consists of 28 items rated on a 5-point Likert scale, with higher scores indicating more severe experiences of abuse and neglect during childhood (32). The CTQ has been widely used among Chinese adolescents and has demonstrated good reliability and validity (33). In the present study, the scale demonstrated good internal consistency (Cronbach’s *α* = 0.83).

Adolescent Non-suicidal Self-injury Assessment Questionnaire (ANSAQ): NSSI was measured using the behavioral section of the ANSAQ. The full instrument consists of a behavioral and a functional subscale; in this study, only the behavioral subscale was used. This subscale includes 12 items assessing the frequency of various self-injurious behaviors without suicidal intent over the past year, rated on a 5-point scale from 0 (“never”) to 4 (“always”), with higher scores indicating more severe NSSI (34, 35). The ANSAQ has been widely applied in adolescent samples and has shown good psychometric properties. In the present sample, the behavioral subscale demonstrated excellent internal consistency (Cronbach’s *α* = 0.91).

Center for Epidemiologic Studies Depression Scale (CES-D): Depressive symptoms were assessed using the CES-D. The CES-D was originally developed by Radloff for use in epidemiologic studies of the general population to assess the frequency and severity of depressive symptoms, rather than solely for clinical diagnosis (36). The scale comprises 20 items rated on a 4-point scale, with higher total scores reflecting more severe depressive symptomatology. Extensive research has shown that the CES-D exhibits good reliability and validity across diverse community and clinical samples and is widely used as a screening tool and severity measure for depressive symptoms (37–40). In the present study, the CES-D demonstrated excellent internal consistency (Cronbach’s *α* = 0.92).

Teacher Caring Scale: Given that students’ own perceptions may more accurately reflect the impact of teacher behavior on their psychological adjustment, perceived teacher care was measured using the Teacher Caring Scale developed by Teven and McCroskey (41). The scale consists of 10 items, with higher total scores indicating higher levels of perceived teacher care. A translation–back-translation procedure was used to ensure linguistic and conceptual equivalence: two doctoral students and two master’s students in psychology independently translated and refined the items into Chinese, after which a university foreign language teacher with a certified advanced translation qualification back-translated the Chinese version into English. Discrepancies were discussed and resolved by the research team to ensure the quality of the final Chinese version. In the present sample, the teacher care scale demonstrated good internal consistency (Cronbach’s *α* = 0.91).To further examine the construct validity of the Teacher Caring Scale in the present sample, confirmatory factor analysis (CFA) was conducted. The results showed acceptable model fit (*χ²/df* = 32.77, CFI = 0.93, TLI = 0.91, SRMR = 0.03), supporting the structural validity of the scale in the current study.

### 2.3 Data analysis

Data were processed and analyzed using SPSS 26.0 to examine the relationships among ACEs, depressive symptoms, perceived teacher care, and NSSI. The total NSSI score was treated as a continuous outcome, and hierarchical multiple regression analyses were conducted. In Step 1, demographic and family variables (e.g., gender and grade) were entered as covariates. In Step 2, ACEs and depressive symptoms were added to test their independent associations with NSSI. In Step 3, teacher care was entered to examine whether it, as a school-based protective factor, was associated with lower NSSI levels. To test the buffering hypothesis, continuous predictors were mean-centered and interaction terms (ACEs × teacher care and depressive symptoms × teacher care) were created and entered into the regression models to determine whether teacher care moderated the associations between ACEs and NSSI and between depressive symptoms and NSSI. Subsequently, moderated indirect effects were tested using the PROCESS macro developed by Hayes (42): Model 4 was used to evaluate the indirect effect of depressive symptoms in the association between ACEs and NSSI, and Model 59 was used to test the moderating effect of teacher care on this indirect pathway. To improve interpretability and analytic precision, variables were standardized prior to model estimation. When interaction effects were significant, simple slope analyses were conducted to compare the ACEs–NSSI and depressive symptoms–NSSI associations at different levels of teacher care. Because all data in the present study were collected through questionnaire surveys, common method bias may be a concern. Therefore, Harman’s single-factor test was conducted to assess common method bias. The results showed that 10 factors had eigenvalues greater than 1, and the first unrotated factor accounted for 23.27% of the total variance, which was below the critical threshold of 40%, indicating that common method bias was not a serious concern in the present study. During data cleaning, invalid questionnaires were excluded based on predefined criteria, and the remaining missing data were handled using listwise deletion. To examine the heterogeneity of the sample across school contexts, one-way analysis of variance (ANOVA) was conducted to test whether the main study variables differed among the three participating school types.

## 3 Results

### 3.1 Correlations among study variables

Spearman rank correlation analyses were conducted for the main study variables (**Table 1**). Teacher care was significantly negatively correlated with ACEs, depressive symptoms, and NSSI, whereas NSSI was significantly positively correlated with both ACEs and depressive symptoms. Because the correlation between ACEs and depressive symptoms was relatively high (*ρ* = .60), collinearity diagnostics were further conducted. The result showed that the variance inflation factor (VIF) was 1.00, indicating that serious multicollinearity was not present. These findings suggest that, in the present sample, ACEs and depressive symptoms were associated with higher levels of NSSI, whereas teacher care was associated with lower levels of NSSI.

**Table 1:** Correlation matrix among main study variables(n=2221).

Main study variables	*M*	*SD*	1	2	3	4
Adverse childhood experiences	1.57	0.42	1			
Depressive symptoms	1.68	0.52	0.60**	1		
Teacher care	4.95	1.10	-.37**	-.34**	1	
Non-suicidal self-injury	1.15	0.40	0.50**	0.54**	-.22**	1

*p <.05, **p <.01, ***p <.001.

### 3.2 Test of the mediating effect of depressive symptoms

Mediation effects were tested using Model 4 in the PROCESS macro (version 4.1) developed by Hayes (42), and the results are presented in **Table 2**. After controlling for gender, grade, only-child status, parental marital status, parental expectations, and satisfaction with academic performance, adverse childhood experiences were significantly associated with non-suicidal self-injury (NSSI) (*β* = 0.49, *t* = 23.91, *p* <.001). In Model 4, gender, parental marital status, and students’ satisfaction with academic performance were significantly associated with NSSI, whereas the other control variables were not significant. After depressive symptoms were entered into the model, the association between ACEs and NSSI remained significant but was attenuated (*β* = 0.26, *t* = 10.77, *p* <.001). In addition, ACEs were significantly associated with depressive symptoms (*β* = 0.59, *t* = 33.03, *p* <.001), and depressive symptoms were also significantly associated with NSSI (*β* = 0.39, *t* = 15.77, *p* <.001). Therefore, Hypothesis 1 was supported, suggesting that depressive symptoms may represent an important affective risk pathway linking ACEs to NSSI.

**Table 2:** Partial mediating effect of depressive symptoms between ACEs and NSSI.

Regression equation		Model fit indices			Coefficient significance		
Outcome variable	Predictor variable	*R*	*R^2^*	*F*	*β*	*t*	*95%CI*
NSSI		0.51	0.26	73.30			
	ACEs				0.49	23.91***	[0.45,0.53]
Depressive symptoms		0.66	0.44	166.90			
	ACEs				0.59	33.03***	[0.55,0.62]
NSSI		0.59	0.34	99.42			
	Depressive symptoms				0.39	15.77***	[0.34,0.44]
	ACEs				0.26	10.77***	[0.21,0.31]

NSSI, non-suicidal self-injury; ACEs, adverse childhood experiences.

*p <.05, **p <.01, ***p <.001.

### 3.3 Examination of the moderating role of teacher care

Moderation effects were tested using Model 59 in the PROCESS macro (version 4.1) developed by Hayes (42), and the results are presented in **Table 3**. After controlling for gender, grade, only-child status, parental marital status, parental expectations, and students’ satisfaction with academic performance, the interaction between ACEs and teacher care in predicting depressive symptoms was not significant (*β* = −0.01, *t* = −0.47, *p* >.05). In contrast, the interaction between depressive symptoms and teacher care in predicting NSSI was significant (*β* = −0.04, *t* = −2.07, *p* <.05), and the interaction between ACEs and teacher care in predicting NSSI was also significant (*β* = −0.17, *t* = −8.63, *p* <.001). In Model 59, gender and parental marital status remained significantly associated with NSSI, whereas the remaining control variables were not significant. These results indicate that teacher care moderates the associations of depressive symptoms with NSSI and of ACEs with NSSI.

**Table 3:** Moderating role of teacher care.

Regression model		Model fit indices			Coefficient estimates		
Outcome variable	Predictor variable	*R*	*R^2^*	*F*	*β*	*t*	*95%CI*
Depressive symptoms		0.67	0.45	156.62			
	ACEs				0.54	27.80^***^	[0.51,0.58]
	teacher care				-0.11	-5.69^***^	[-0.14,-0.07]
	ACEs×teacher care				-0.01	-0.47	[-0.03,0.02]
NSSI		0.64	0.40	108.02			
	ACEs				0.18	7.54^***^	[0.14,0.23]
	Depressive symptoms				0.38	15.76^***^	[0.33,0.43]
	teacher care				0.00	0.06	[-0.04,0.04]
	ACEs×teacher care				-0.17	-8.63^***^	[-0.20,-0.13]
	Depressive symptoms×teacher care				-0.04	-2.07^*^	[-0.08,-0.00]

*p <.05, **p <.01, ***p <.001.

**Figure 2:**
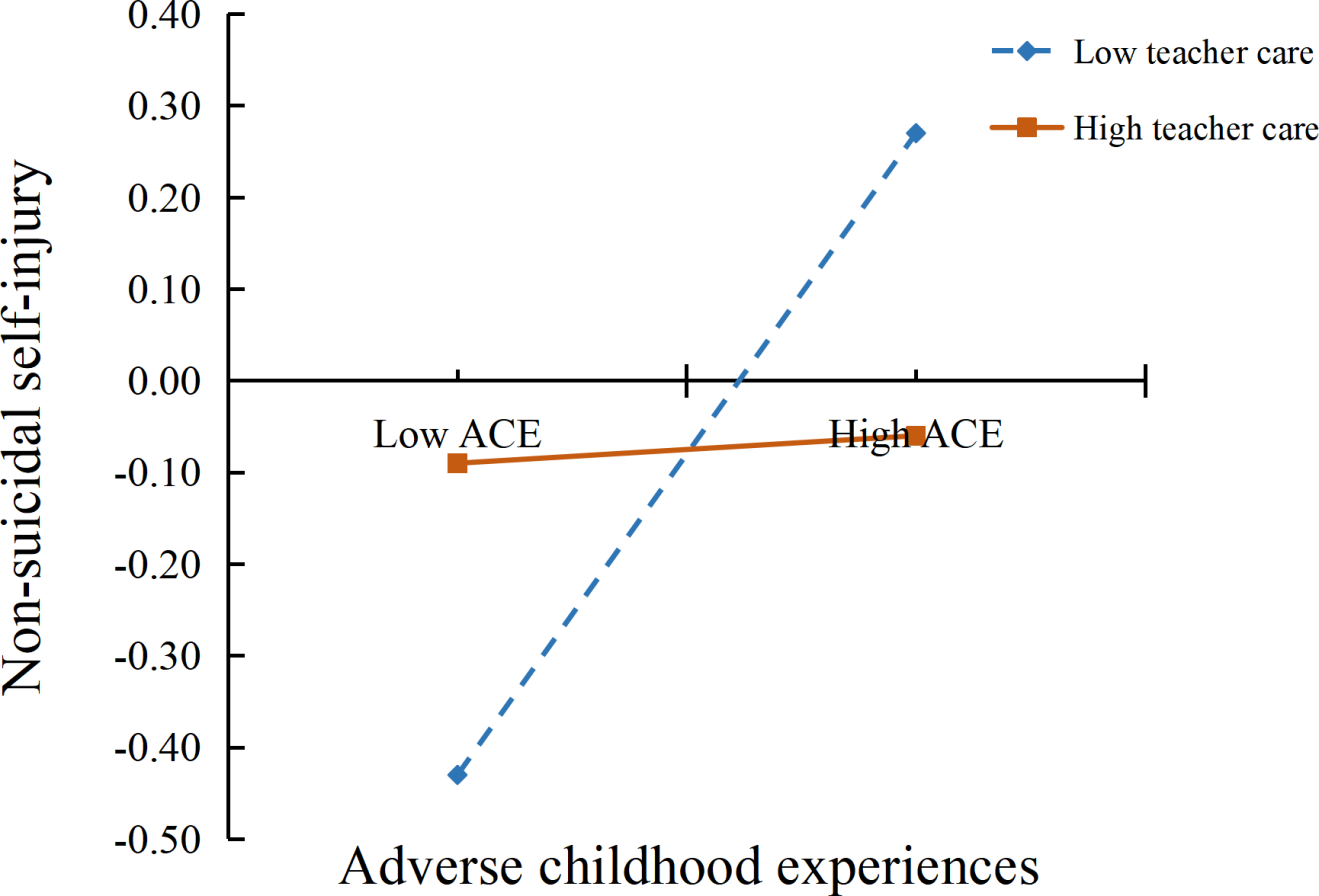
Moderating effect of teacher care on the association between adverse childhood experiences and non-suicidal self-injury.

**Figure 3:**
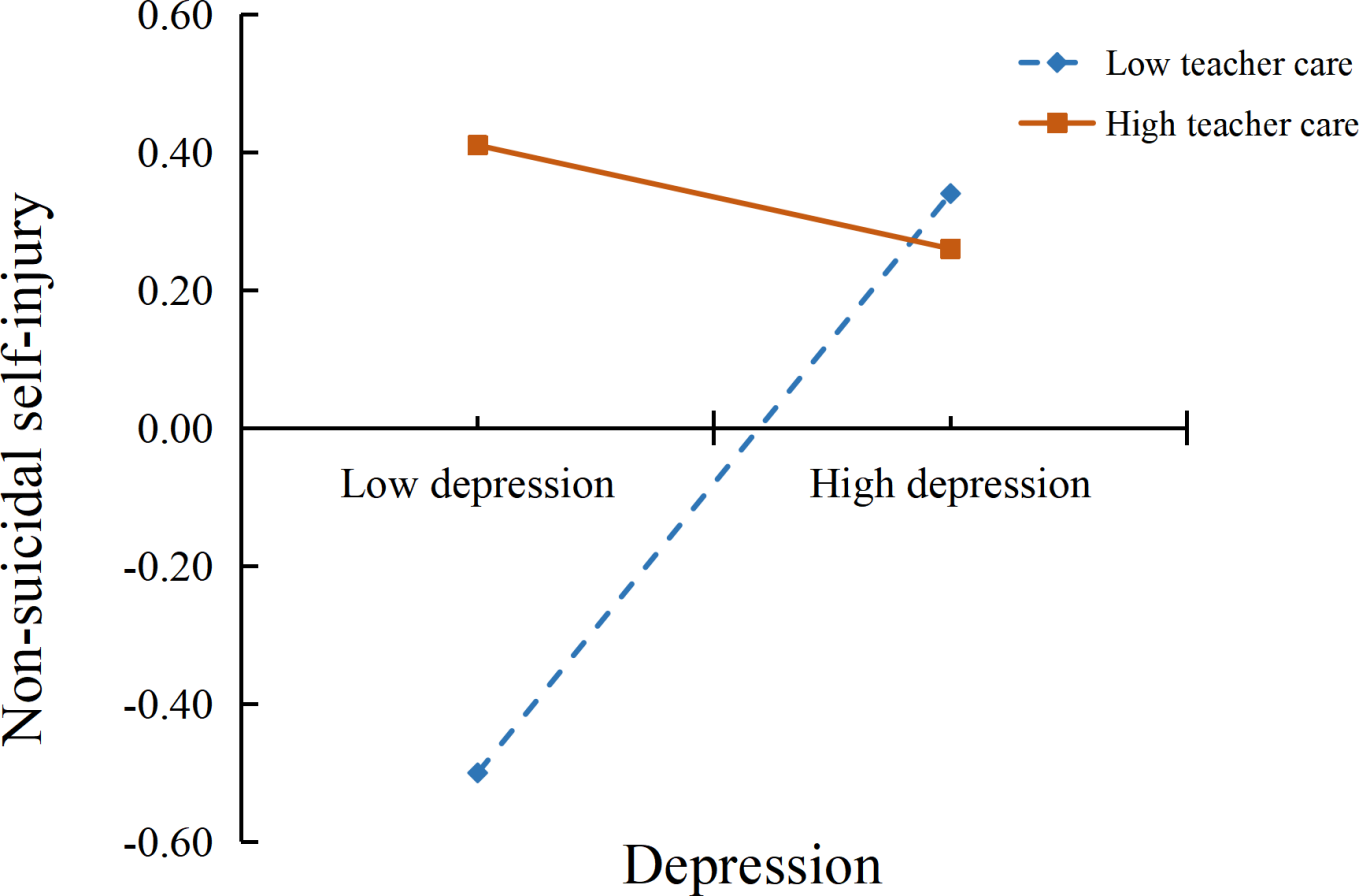
Moderating effect of teacher care on the association between depressive symptoms and non-suicidal self-injury.

To illustrate the moderated mediation model more clearly, we defined high and low teacher care using the mean ± 1 standard deviation and conducted simple slope analyses to examine the moderating effects of teacher care on the ACEs → NSSI and depressive symptoms → NSSI paths. The specific moderation patterns are shown in **Figure 2** and **3**. The results indicated that among students perceiving low teacher care, higher levels of ACEs were associated with more severe NSSI (*β* = 0.35, *t* = 12.80, *p* <.001), whereas this association was not significant among students perceiving high teacher care (*β* = 0.02, *t* = 0.51, *p* >.05). Similarly, among students perceiving low teacher care, higher depressive symptoms were associated with more severe NSSI (*β* = 0.42, *t* = 14.19, *p* <.001). Among students perceiving high teacher care, higher depressive symptoms were associated with lower NSSI (*β* = −0.34, *t* = −9.91, *p* <.001). Therefore, Hypothesis 2 was supported.

## 4 Discussion

In recent years, adolescent mental health has become a major public concern worldwide. Given its high prevalence and serious consequences, non-suicidal self-injury (NSSI) has emerged as one of the most pressing issues in this field (43). Grounded in developmental psychopathology and a social-ecological perspective, the present study examined the relationships among adverse childhood experiences (ACEs), depressive symptoms, teacher care and NSSI in a sample of Chinese adolescents. We found that ACEs and depressive symptoms were significantly associated with higher levels of NSSI, whereas teacher care was associated with lower NSSI levels. After controlling for demographic and family variables, ACEs and depressive symptoms remained important risk factors for NSSI, while teacher care continued to function as a protective factor in the school context. In addition, the positive associations between ACEs and NSSI and between depressive symptoms and NSSI were markedly attenuated at higher levels of teacher care. Overall, from a risk–protective factor perspective, the findings outline a basic “ACEs–depression–teacher care–NSSI” pattern, providing new empirical evidence for understanding the mechanisms underlying adolescent NSSI and offering important implications for prevention and intervention efforts. Compared with prior studies that mainly emphasized family- and individual-level risks, the present study contributes to the literature by integrating perceived teacher care into the ACEs–depressive symptoms–NSSI framework and by providing evidence from a Chinese adolescent sample, thereby extending understanding of school-based protection in a non-Western cultural context.

### 4.1 ACEs and NSSI: The role of distal risk factors

The present study showed that ACEs were moderately positively associated with NSSI in adolescence: even after adjusting for gender, grade and other covariates, higher ACEs were linked to higher NSSI levels. This finding is consistent with prior research (5, 11), indicating that early experiences of abuse, neglect and other adversities can exert long-lasting effects on adolescents’ psychological and behavioral development. Reducing exposure to adverse childhood experiences and enhancing social support may therefore help lower the risk of NSSI (44).According to Nock’s integrated model of self-injury, early adverse experiences can undermine individuals’ capacities for emotion regulation and interpersonal adaptation, thereby increasing the likelihood of using self-injury as a coping strategy when confronted with stressors (1, 14). Adolescence itself is a highly sensitive period for psychological development, during which maladaptive memories and negative emotions associated with early trauma may be readily reactivated, potentially triggering NSSI. Our findings further confirm that ACEs function as important distal risk factors for adolescent NSSI and support the notion within the integrated model that childhood trauma serves as a distal vulnerability for self-injury. At the same time, the results are consistent with Yates’s developmental psychopathology framework, which conceptualizes NSSI as a regulatory behavior that partially compensates for developmental disruptions and mitigates the impact of early adaptation failures (13). Evidence from this school-based, non-clinical sample aligns well with these theoretical perspectives and underscores the importance of systematically assessing adverse childhood experiences when evaluating adolescents’ emotional and behavioral problems.

### 4.2 Depressive symptoms and teacher care: emotional risk, school-based protection, and cross-cultural implications

The present study further showed that depressive symptoms were not only significantly positively associated with NSSI, but also statistically partially accounted for the association between ACEs and NSSI: adolescents with higher levels of ACEs reported more severe depressive symptoms, and higher depressive symptom levels, in turn, were associated with higher NSSI levels. Although the cross-sectional design precludes firm conclusions about temporal ordering, this pattern is consistent with the experiential avoidance model of NSSI (20), which proposes that individuals with impaired emotion regulation capacities may engage in NSSI to escape or reduce internal distress. Prior work has likewise suggested that adolescents who experience depressive affect and lack effective coping strategies often resort to NSSI as a means of obtaining immediate psychological relief (29), and that NSSI can become a repeatedly used coping strategy when adolescents confront emotional crises (18, 22).Childhood maltreatment has also been identified as a distal factor for later depression, with individuals exposed to early trauma more likely to exhibit pronounced depressive symptoms (45). In our analyses, when depressive symptoms were included, the association between ACEs and NSSI remained significant, suggesting that depression statistically explains part, but not all, of this association. These findings further support and extend emotion regulation perspectives on NSSI. On the one hand, traumatic experiences in childhood are often accompanied by enduring negative emotional responses, particularly depression, which may undermine emotion regulation capacities and make adolescents more likely to use NSSI as a maladaptive strategy to alleviate emotional pain. On the other hand, such adverse experiences may erode adolescents’ ability to build positive emotional experiences and social connections, thereby exacerbating depressive symptoms and increasing the risk of NSSI. Consistent with this, previous research has shown that mindfulness and depressive symptoms jointly mediate the association between ACEs and NSSI, highlighting the importance of negative affective pathways in this process (46). Taken together, depression, as a relatively stable negative emotional state, appears to be a key psychological mechanism linking childhood trauma and NSSI, and emotional disruptions associated with ACEs may manifest during adolescence as elevated depressive symptoms that, in turn, heighten NSSI risk. These findings provide empirical support for NSSI prevention approaches that target affective disorders as core intervention foci.

At the same time, this study examined teacher care as a protective resource within the school context. Teacher care moderated both the direct association between ACEs and NSSI and the association between depressive symptoms and NSSI. Under conditions of low teacher care, the impact of ACEs on NSSI was markedly stronger, and the association between depressive symptoms and NSSI was tighter; in contrast, at higher levels of teacher care, the strengths of both associations were substantially reduced, indicating a buffering role of teacher care in risk pathways. This pattern is consistent with the risk buffering hypothesis, which posits that when individuals are exposed to adverse environments, stable social support can significantly attenuate the negative impact of risk factors on psychological and behavioral outcomes. As a key source of social support in the school environment, teachers’ caring behaviors can help adolescents build positive interpersonal relationships, strengthen their sense of school belonging and self-efficacy, and thereby enhance overall mental health. The buffering role of teacher care in the association between ACEs and NSSI may be understood from a developmental psychopathology and risk-buffering perspective. Adolescents with a history of adverse childhood experiences may be more vulnerable to emotional insecurity, maladaptive coping tendencies, and disrupted interpersonal expectations, all of which may increase the likelihood of engaging in NSSI (12–14). In this context, teacher care may function as a compensatory relational resource within the school environment. By providing emotional support, consistency, guidance, and a sense of interpersonal safety, caring teachers may help reduce the extent to which early adversity is associated with self-injurious behavior (47). Teacher care may therefore not only coexist with lower NSSI levels, but may also weaken the relational and emotional conditions under which ACE-related vulnerability becomes behaviorally expressed through NSSI. Previous studies have shown that students who receive more support from teachers are less likely to engage in NSSI (47). Warm teacher–student relationships and a strong sense of school belonging are core features of a supportive school climate, which can promote students’ academic, emotional and social development, buffer the psychosocial risks associated with ACEs, and reduce the likelihood of NSSI and suicidal behavior (30). Teacher care has also been linked to lower depressive symptoms (23) and better school adjustment and emotion regulation. On the one hand, teacher care may help adolescents cope more effectively with negative emotions and interpersonal stress through emotional support, positive feedback and a sense of safety, thereby reducing their reliance on NSSI. On the other hand, teachers’ ongoing observation and daily contact with students may facilitate earlier detection of emotional distress and risk behaviors, enabling timely support and referral. By placing teacher care alongside ACEs and depressive symptoms within the same analytic framework, the present study illustrates how risk and protective factors jointly operate in the same school context, and provides new empirical evidence for understanding school-level protective mechanisms in relation to adolescent NSSI.

The present study may also contribute to cross-cultural psychology research. Although NSSI is a global mental health issue, its psychosocial processes may vary across cultural contexts. In the Chinese context, teachers are often expected to provide not only academic instruction but also emotional and moral support. Therefore, perceived teacher care may represent a culturally salient protective resource. From a cross-cultural perspective, the present findings suggest that while the risk pathways linking ACEs, depressive symptoms, and NSSI may be broadly shared, the buffering role of protective factors may depend on culturally embedded relationship structures. In this sense, the study adds evidence from a non-Western setting and helps bridge universal and culture-specific perspectives in adolescent mental health research.

### 4.3 Methodological limitations and directions for future research

Guided by a developmental psychopathology framework and drawing on the integrated model of NSSI and the experiential avoidance model, the present study examined associations among ACEs, depressive symptoms, teacher care and NSSI in Chinese adolescents from a risk–protective factor perspective. While the findings add to the literature, several methodological limitations warrant cautious interpretation. First, the study used a cross-sectional design and all variables were assessed at a single time point via self-report, which limits our ability to determine the temporal ordering among ACEs, depressive symptoms and NSSI. Although, at the conceptual level, ACEs occur in childhood and depressive symptoms and NSSI typically emerge later in adolescence, the present results should be interpreted statistically as supporting an “ACEs–depression–NSSI” risk pathway in terms of associations, rather than as providing direct evidence for a causal chain. Because ACEs were measured using the CTQ, the present findings mainly reflect maltreatment-related adversity rather than the full spectrum of ACE domains. Future research would benefit from longitudinal designs in which ACEs, depressive symptoms and NSSI are assessed at multiple time points. Approaches such as cross-lagged panel models or growth curve models could then be used to more rigorously test temporal precedence and the dynamic evolution of these variables. Second, the study relied primarily on adolescents’ self-report questionnaires, which may be subject to recall bias and social desirability effects. This is particularly relevant when retrospectively reporting childhood experiences, as current emotional states may influence memories and evaluations of past events. It is also important to note that the buffering role of teacher care may be interpreted in more than one way. On the one hand, adolescents in high-care school environments may develop stronger perceived relational expectations and therefore become more likely to respond in socially desirable ways, potentially underreporting stigmatized behaviors such as NSSI (48, 49). On the other hand, teacher care may genuinely reshape the coping processes of adolescents with depressive symptoms by providing emotional validation, interpersonal security, and more adaptive regulatory resources, thereby reducing reliance on self-injury as a coping strategy (23, 24, 26). These two interpretations are not mutually exclusive. However, because the present study relied on cross-sectional self-report data, it was not possible to determine whether the observed pattern mainly reflected reporting tendencies, actual coping changes, or both. Subsequent research could incorporate multiple informants and multi-method data (e.g., parent or teacher reports, interview data, and, when feasible, more objective indicators) to reduce common method bias and strengthen the robustness of the findings. Third, the sample was drawn from three schools in central China. Although it included an urban key school, a school for children of rural-to-urban migrant workers and a rural school, the geographic and cultural diversity of the sample remains limited. Future studies should expand sampling to include adolescents from different regions, developmental stages (e.g., late primary school, senior high school) and cultural backgrounds in order to examine the generalizability and potential cultural specificity of the present findings. Finally, this study focused on three sets of variables—ACEs, depressive symptoms and teacher care—within the NSSI risk structure. However, the broader risk network for NSSI likely also involves factors such as anxiety, impulsivity, family functioning, peer relationships and cyberbullying. Future research could incorporate a broader array of risk and protective indicators in larger samples and use methods such as network analysis, structural equation modeling or machine learning to identify key nodes and high-risk constellations. Such work would help to develop more fine-grained maps for the precise identification of risk and the tailoring of interventions.

### 4.4 Practical implications: school-based interventions from a risk–protective factor perspective

Despite these limitations, the present findings offer several implications for school-based NSSI prevention and intervention. First, with respect to risk identification, the results highlight the need to pay particular attention to students who show both high levels of ACEs and marked depressive symptoms. These adolescents occupy a “disadvantaged combination” position within the NSSI risk network and may be especially likely to develop self-injurious behaviors. School mental health screening could appropriately include brief assessments of early adverse experiences and depressive symptoms, and combine these with teachers’ observations and parental reports to establish a multi-channel system for identifying at-risk students. Second, in terms of intervention priorities, the findings support targeting depression and other negative emotional states as key intervention foci. Helping adolescents with a history of adverse childhood experiences (ACEs) strengthen emotion regulation and coping strategies may reduce their tendency to use NSSI as an emotional outlet. School-based mental health services can include group counseling, individual counseling and psychoeducational programs that teach emotion recognition and regulation skills, while providing more intensive psychological support for high-risk individuals. Third, the study underscores the pivotal role of teacher care within the broader risk–protection structure, suggesting that in NSSI prevention and intervention, teachers are not only “detectors of problems” but also an integral part of the protective mechanism. Schools may provide teachers with training in trauma-informed education, supportive communication skills, emotional validation, and the early recognition of warning signs related to depressive distress and self-injurious behavior. Such training may help teachers develop a deeper understanding of how early adversity and emotional difficulties are reflected in students’ behavior and adjustment within the school context. Caring classroom practices may also be promoted through everyday interactions. For example, teachers may strengthen regular teacher–student communication, provide empathic and respectful feedback, maintain predictable and supportive classroom management, and establish safe communication channels through which students can seek help when needed. These practices may enhance students’ sense of interpersonal safety, belonging, and support, thereby reducing the likelihood that emotional distress is managed through self-injury. In addition, schools may consider integrating teacher care into teacher development programs, school mental health policies, and classroom evaluation systems, so that caring practices become part of routine educational work rather than relying solely on individual teachers’ personal awareness. In this way, strengthening teacher care may be translated into practical relational strategies and organizational support measures. Fourth, at the policy and systems level, the present findings support integrating “reducing early adverse experiences” and “strengthening supportive school environments” into broader plans for youth mental health promotion and suicide prevention. Education and health agencies could work together to advance child protection, family support and school mental health services, embedding ACEs risk screening, depression identification and enhancement of teacher care within a unified policy framework. Building such a multi-level network of risk and protective factors may help to more systematically reduce the population burden of NSSI and related affective disorders among adolescents.

## 5 Conclusions

In summary, the present study yielded three main findings. First, adverse childhood experiences (ACEs) were significantly positively associated with adolescent non-suicidal self-injury (NSSI), and remained an important distal risk factor for NSSI after controlling for demographic and family variables. Second, depressive symptoms were strongly related to NSSI and partially accounted for the association between ACEs and NSSI, suggesting that emotional difficulties associated with early adverse experiences may be reflected in elevated depressive symptoms during adolescence, which were also associated with higher levels of self-injury. Third, teacher care was associated with lower levels of NSSI and played a buffering role within the broader risk–protective structure: among adolescents who perceived higher teacher care, the positive associations between ACEs and NSSI and between depressive symptoms and NSSI were markedly attenuated. Taken together, these findings indicate that, alongside addressing risk factors such as ACEs and depression, systematically enhancing teacher care in the school context may be crucial for reducing the risk of NSSI among adolescents.

## Data Availability

The raw data supporting the conclusions of this article will be made available by the authors, without undue reservation.
